# Anti-PD-1 Immunotherapy and Radiotherapy for Stage IV Intrahepatic Cholangiocarcinoma: A Case Report

**DOI:** 10.3389/fmed.2020.00368

**Published:** 2020-08-28

**Authors:** Ze-Long Liu, Xin Liu, Hong Peng, Zhen-Wei Peng, Jian-Ting Long, Di Tang, Sui Peng, Yong Bao, Ming Kuang

**Affiliations:** ^1^Division of Interventional Ultrasound, The First Affiliated Hospital of Sun Yat-sen University, Guangzhou, China; ^2^Department of Liver Surgery, The First Affiliated Hospital of Sun Yat-sen University, Guangzhou, China; ^3^Clinical Trials Unit, The First Affiliated Hospital of Sun Yat-sen University, Guangzhou, China; ^4^Department of Radiotherapy, The First Affiliated Hospital of Sun Yat-sen University, Guangzhou, China; ^5^Department of Oncology, The First Affiliated Hospital of Sun Yat-sen University, Guangzhou, China; ^6^Department of Gastroenterology, The First Affiliated Hospital of Sun Yat-sen University, Guangzhou, China

**Keywords:** intrahepatic cholangiocarcinoma, immunotherapy, radiotherapy, combination therapy, biomarkers

## Abstract

Due to the unsatisfactory robustness of current predictive biomarkers in many cases, application of immunotherapy in advanced cancers with limited treatment options, such as stage IV intrahepatic cholangiocarcinoma (ICC), was quite common. Hence, strategies to enhance the therapeutic effect of immunotherapy or to extend the scope of potential beneficial patients were urgently needed. Combination of radiotherapy and anti-programmed death receptor-1 (PD-1) immunotherapy was a promising one, since they were found to have a synergistic anti-tumor effect in animal models and a couple of patients. We here present a 68-years-old male with chemotherapy-intolerable stage IV ICC, whose primary tumor had low PD-L1 expression level, scarce CD8+ cells in tumor microenvironment, high microsatellite instability (MSI), and high tumor mutation burden (TMB). These biomarkers showed a conflicting prediction of the treatment response and clinical benefit of anti-PD-1 immunotherapy. Combination therapy of anti-PD-1 immunotherapy and radiotherapy was adopted as first-line treatment for the patient. After six cycles of immunotherapy, shrinkage of the primary liver tumor and metastatic lymph nodes happened, alongside with new lung metastasis, which indicated a mixed response. Radiotherapy was then administered to both the liver and lung lesions, accompanied with continued immunotherapy. The combined therapy eventually led to a complete response for both the primary tumor and all metastases without treatment-related adverse effects. The patient has survived for 26 months after the combined therapy and remains tumor-free currently. This case demonstrates the high inconsistency between immunotherapy response biomarkers and the synergetic anti-tumor effect of immunotherapy and radiotherapy in ICC.

## Introduction

Stage IV intrahepatic cholangiocarcinoma (ICC) patients have very poor survival outcomes. Gemcitabine plus cisplatin chemotherapy is currently recommended as the only first-line treatment for these patients, with a median overall survival (OS) of only 11.7 months (1). The worst is that more than 70% of patients are intolerable to the chemotherapy regimen because of severe complications. Therefore, the use of current chemotherapy for most stage IV ICC patients is limited and the requirement for a novel treatment option is urgent (1).

Recently, immune checkpoint blockades showed promising therapeutic effects in a wide range of solid tumors, including a small number of ICC cases (2). However, robust biomarkers for predicting treatment response remains one of the most crucial issues. Although several biomarkers including PD-L1 expression level, microsatellite instability (MSI), tumor mutation burden (TMB), and immune cell infiltration have been applied for selecting target patients, their accuracies were all limited and diverse across different types of tumors. Only MSI was reported to be predictive in a few ICC cases (2). On the other hand, general outcomes of anti-PD-1 immunotherapy for ICC remain controversial. Thus, considering the lack of robust biomarkers and the limited treatment options for cholangiocarcinoma, it is more urgent to find out universal strategies for applying immunotherapy. Most evidence by far shows the inadequate efficacy of immunotherapy alone for the control of advanced cancer.

Radiotherapy is another treatment option for unresectable ICC, which showed a local control effect (3, 4). However, due to limited evidence, recommendations of anti-PD-1 immunotherapy and radiotherapy are both category 2A. It has been reported that local tumor destruction combined with immunotherapy may have a synergetic effect against solid tumors (5). Radiotherapy is a powerful local treatment that can only reduce tumor burden to the minimal but also trigger the anti-tumor immunity and reprogram the tumor microenvironment. Yet, present evidence of the synergistic anti-tumor effect of radiotherapy and immunotherapy for ICC is lacking.

Here we comprehensively investigated the current predictive markers and showed their inconsistency and complexity in a chemotherapy-intolerable stage IV ICC patient with metastases to lymph nodes and lungs, who had a complete response and survival benefit to the combination therapy of immunotherapy and radiotherapy as the first-line treatment.

## Case Presentation

**Figure 1:**
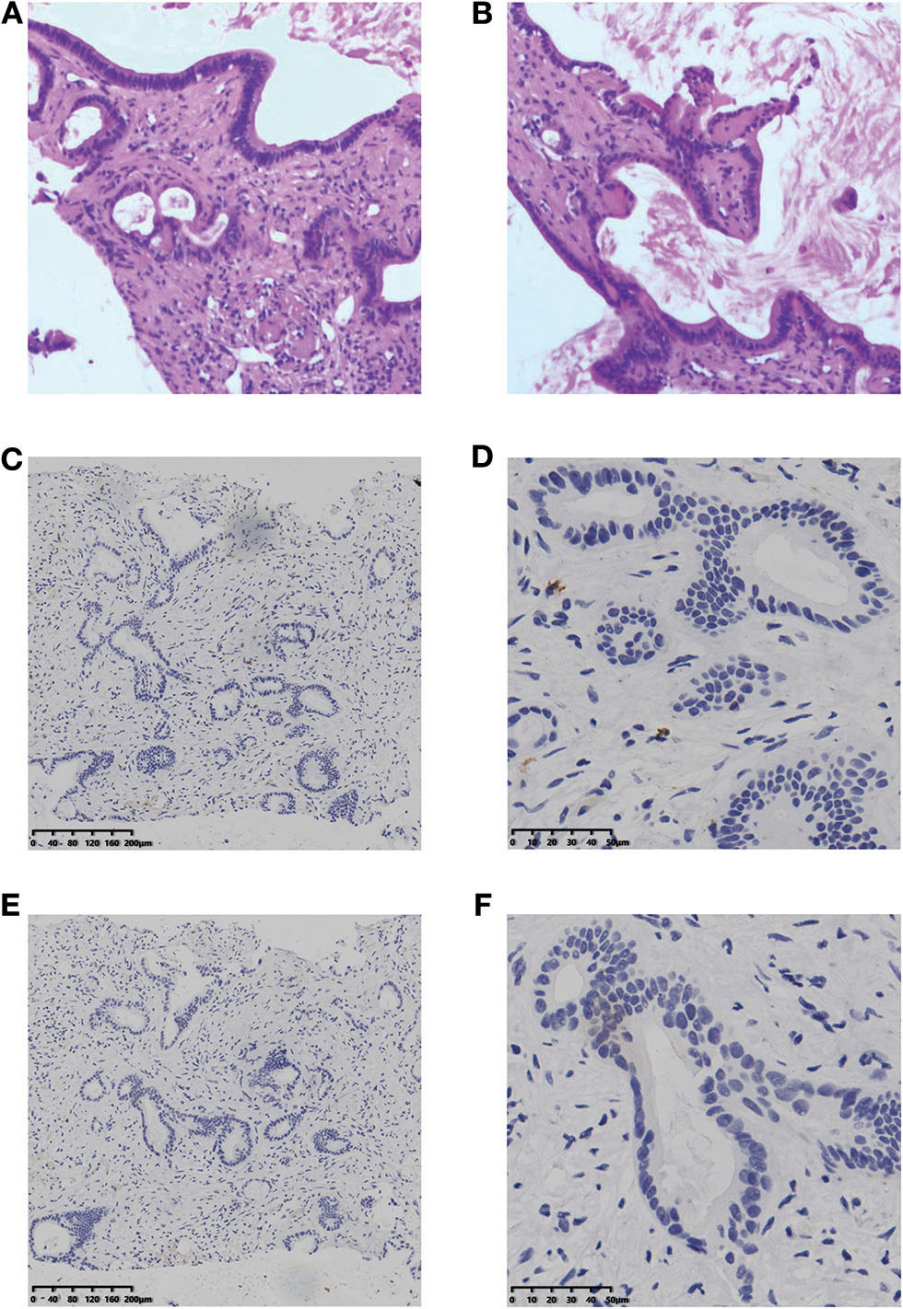
H&E staining **(A, B)** and IHC result of CD8 **(C,D)** and PD-L1 **(E,F)** for the liver lesion. H&E staining, hematoxylin and eosin staining; IHC, immunohistochemistry; PD-L1, programmed cell death ligand 1.

A 68-years-old male complained with xanthochromia, scleral icterus, and abdominal distension for over 20 days was admitted to our hospital in January 2018. He lost about 10 kg of body weight. Physical examination showed deep jaundice of the patient and the left supraclavicular lymph nodes were palpable. The performance status (PS) score was 3. Laboratory tests showed that total bilirubin (TB) was 707.9 umol/L, and CA19-9 level was over 12,000 U/mL, while AFP level was <20 ug/L (Table 1). Magnetic resonance imaging (MRI) found a 47 × 42 mm space-occupying lesion in Segment 4 (S4) and S5 of the liver and a mass in the common bile duct, suspicious for ICC. Subsequent positron emission tomography (PET) showed multiple distant metastases to lungs, abdominal lymph nodes, and left cervical lymph nodes. Histology of the liver lesion biopsy found numerous tubular structures of adenocarcinoma and a fibrous stoma (Figures 1A,B). Immunohistochemistry (IHC) analysis showed the following: CK(+), CK7(+), CK20(weak +), and Ki-67(3%+). The diagnosis was confirmed as stage IV ICC. The presumed survival time was only 3–5 months (6).

**Table 1:** Clinical variables of the patient during treatment.

**Variables**	**22 Jan 2018 (baseline)**	**8 Apr 2018**	**31 Jul 2018**	**10 Dec 2018**	**14 Feb 2019**	**31 May 2019**
Size of the liver lesion (mm × mm)	47 × 42	38 × 33	35 × 29	32 × 23	29 × 21	10 × 7
Total bilirubin (umol/L)	707.9	114.0	21.2	9.7	23.0	12.9
CA199 (U/mL)	>12,000	>12,000	4620.49	109.31	36.41	14.70
CEA (ug/L)	19.69	8.90	2.97	1.39	1.62	1.82
CA125 (U/mL)	114.90	60.10	15.50	12.50	12.70	10.60
AFP (ug/L)	2.20	5.26	3.01	3.84	2.99	3.15

**Figure 2:**
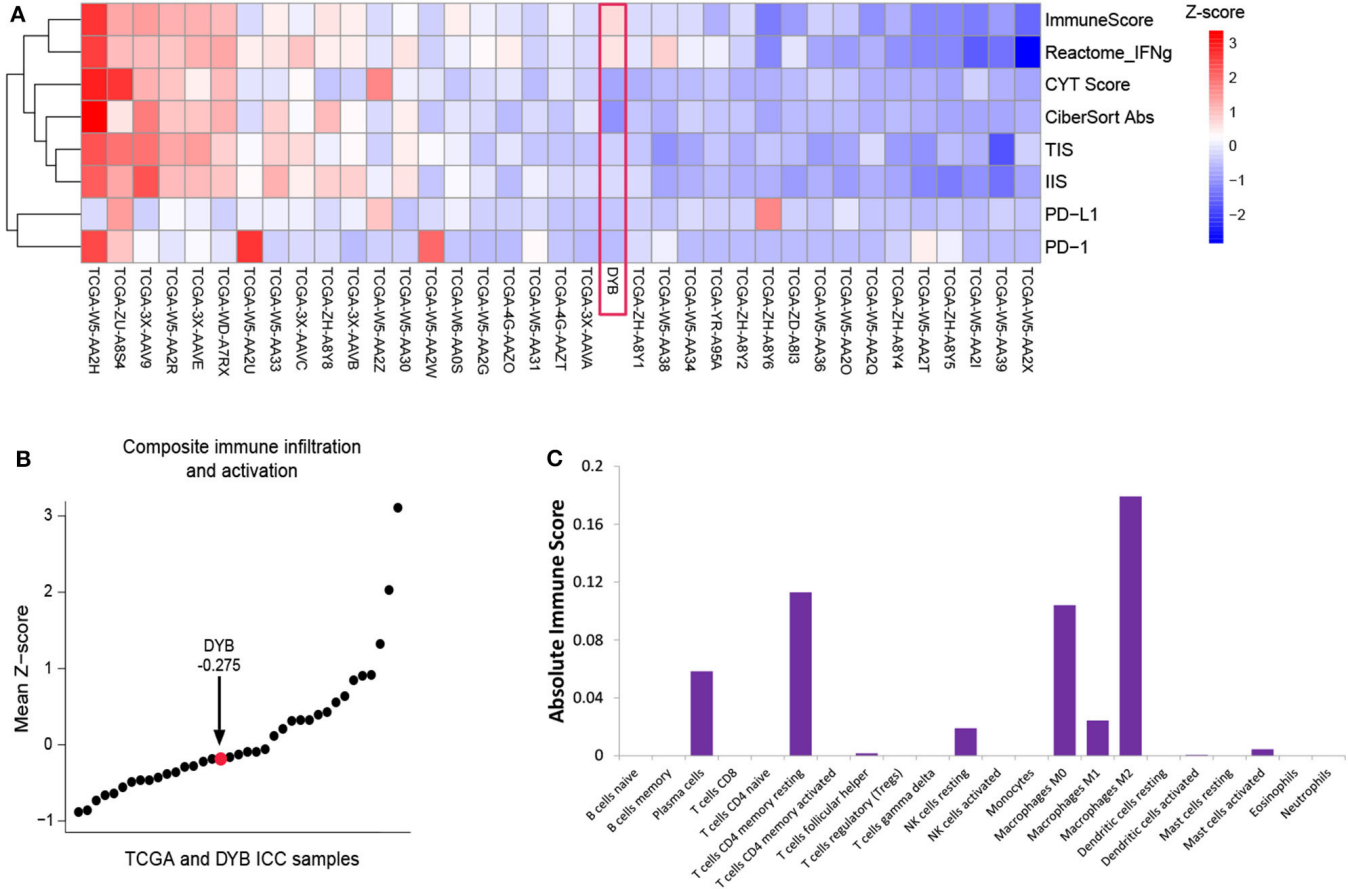
Immune characterization of the primary tumor before treatment. **(A)** The heatmap of the ICC case and 36 ICC cases in the TCGA dataset, with the measurement of ImmunoScore, interferon-γ signaling (Reactome.org), CYT score, Cibersort Absolute Score, TIS, IIS, PD-L1, and PD-1. **(B)** Plot of the mean Z-scores across this ICC case and 36 ICC cases in TCGA dataset. **(C)** The absolute immune score shows the components of the immune cells in primary tumor of the ICC case. ICC, intrahepatic cholangiocarcinoma; TCGA, the Cancer Genome Atlas; TIS, T cell Infiltration Score; IIS, Immune Infiltration Score; PD-L1, programmed cell death ligand 1; PD-1, programmed death receptor-1.

**Figure 3:**
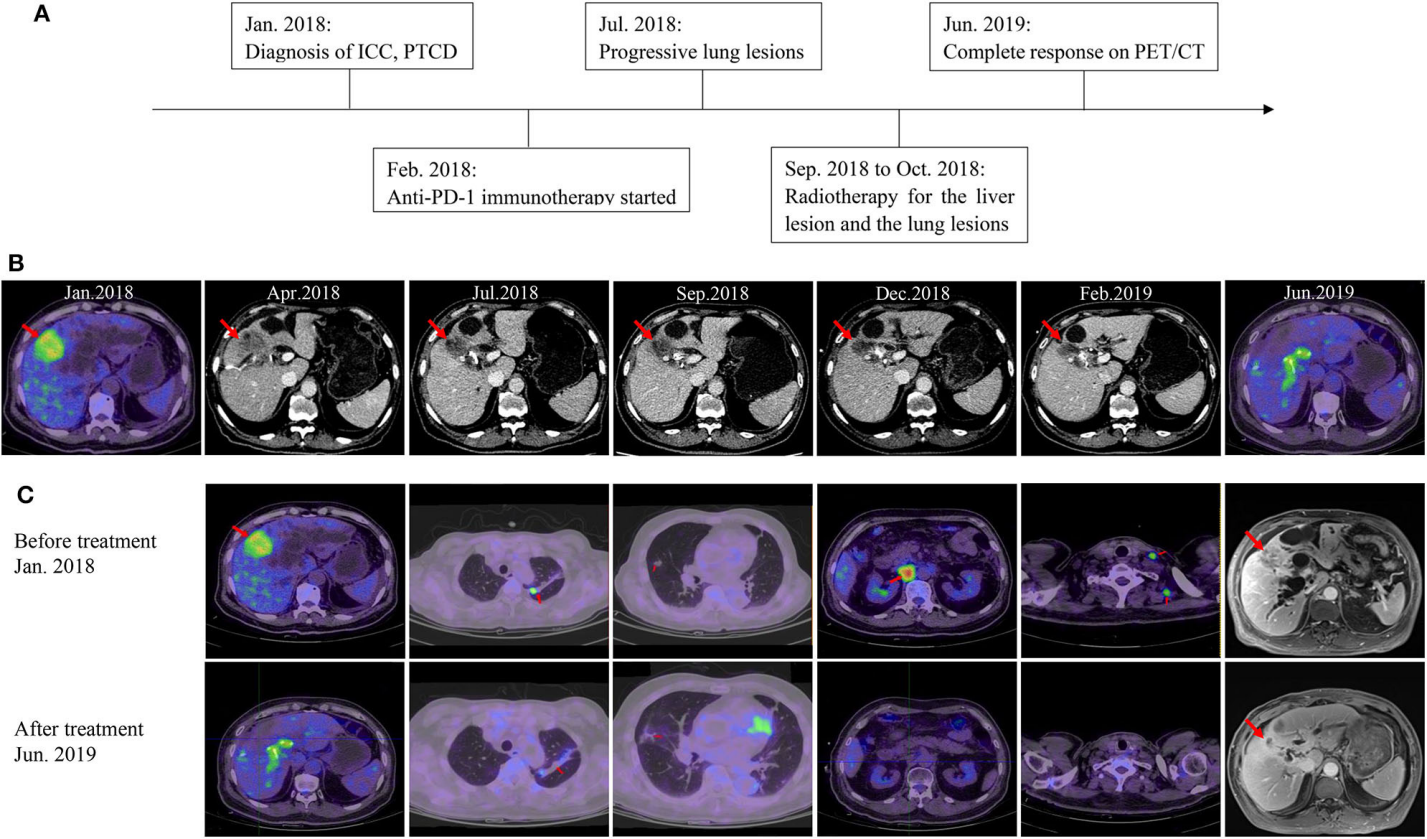
Imaging of the patient during treatment. **(A)** The timeline of his clinical course. **(B)** Imaging shows the change of the liver lesion over time. **(C)** Imaging of the patient at baseline and the latest follow-up.

According to the opinion of the ICC multi-discipline team in our hospital, the patient was not a candidate for conventional treatments including surgery and chemotherapy, considering both tumor and PS status. Then, percutaneous transhepatic cholangial drainage (PTCD) was performed to relieve the jaundice and the patient's appetite recovered and the PS score was still 3. To comprehensively investigate the immune microenvironment, the tumor tissue of the liver lesion was submitted for subsequent tests. Additional IHC analysis found a low expression level of programmed cell death ligand 1 (PD-L1) and a low frequency of CD8+ T cells (Figures 1C–F). The whole-exome sequencing (WES) data showed high levels of both MSI and TMB (16.9 mutations/Mb), which indicated the potential benefit of immunotherapy. Additionally, there were 420 indels (insertions and deletions) and 660 single nucleotide variants (SNVs), with five mutations (including *MLH1, SMARCA4, BRCA2, POLE2*, and *ARID1A*) known to be associated with sensitivity to immunotherapy while one gene (*B2M*) conferred resistance to immunotherapy. We further included another 36 ICC cases in the Cancer Genome Atlas (TCGA) dataset to comparatively analyze the patient's tumor immune microenvironment based on the RNA-seq data. This case was found to have a moderate level of immune infiltration under a comprehensive immune signature (Figures 2A,B) (7–11). Analysis of immune cell components in the tumor microenvironment using the CIBERSORT algorithm revealed scarce CD8+ cells but a large number of M2 macrophages, which is consistent with the IHC result and indicates an immunodeficient state (Figure 2C) (10). After all, anti-PD-1 immunotherapy (pembrolizumab, at a dose of 200 mg every month) combined with radiotherapy was considered as treatment for the patient, which was initiated in February 2018 (Figure 3A).

After two cycles of immunotherapy, the patient's symptoms relieved and his PS improved. The size of the liver lesion slightly reduced to 38 × 33 mm but CA-199 was still over 12,000 U/mL (Figure 3B; Table 1). After six cycles, PS score was 1 and CA-199 was decreased to 4620.49 U/mL (Table 1). Contrast-enhanced computed tomography (CT) scans showed that the liver lesion reduced to 32 × 23 mm (Figure 3B). However, the number of lung metastases increased, which indicated a mixed response to immunotherapy (Figure 3C). Anti-PD-1 immunotherapy continued while radiotherapy was introduced to control the liver and lung lesions, with doses of 50.0 and 48.0 Gy, respectively. All visible tumors reduced in size gradually in the follow-up and the PTCD was removed 3 months later (Figure 3C). Currently, after 26 months of treatment, the patient is alive with high life quality. There aren't any symptoms and PS score is 1. The patient regained 5 kg of body weight. All tumor biomarkers including CA19-9 level are normal. The latest imaging examinations show invisible signs of the liver lesion, the metastatic lymph nodes, and the lung metastases. CR is achieved in this stage IV ICC case (Figure 3C; Table 1).

## Discussion

Currently, emerging evidence shows the therapeutic effect of anti-PD-1 immunotherapy in various types of cancers, yet target patient selection remains one of the biggest problems. Although several biomarkers including PD-L1 expression level, TMB, MSI, or immune cell infiltration, have been used to select patients and predict treatment response in anti-PD-1 immunotherapy, they were still not reliable in many situations. As for cholangiocarcinoma, only weak evidence showed that MSI had the potential to be an appropriate predictive marker. Undoubtedly, the anti-tumor immune response is a very complicated biological process that involved cancer cells and cells in the microenvironment. Each biomarker only reflected some aspect of the whole process and it was no wonder that they would be inconsistent with others and fail to predict in some situations. In this case, we comprehensively analyzed the immune microenvironment of the patient and found that although both MSI and TMB were high, the PD-L1 expression level was low and the immunosuppressive tumor microenvironment of the liver lesion had scarce CD8+ cells but lots of M2 macrophages. High infiltration of M2 macrophages in the tumor stroma could suppress T cell infiltration and down-regulate antitumor immune responses. The contradiction between biomarkers resulted in difficulty in predicting response. Even though both MSI and TMB are currently the most valuable predictive biomarkers for anti-PD-1 immunotherapy, there are also lots of cancer patients with MSI-H or/and TMB-H that do not respond well. According to previous studies, only approximately half of solid tumors with MSI-H achieved object response to anti-PD-1 immunotherapy (2). Besides, low lymph cell infiltration in this case might also indicate immune escape, which allows tumor evolution and thus higher genomic diversity. The tumor with this situation was considered to be unresponsive to immunotherapy (12). On the other hand, tumor heterogeneity also influences the accuracy in determining the status of these markers (13). Intratumor genetic heterogeneity was found obvious in ICC and multi-point aspiration was needed to evaluate the markers accurately, which was impossible in patients that did not receive surgery or underwent tumor recurrence. In a word, there is currently no robust marker for predicting the response to anti-PD-1 immunotherapy. On one hand, further studies are needed to develop robust predictive markers for selecting those patients that might benefit from anti-PD-1 immunotherapy. On the other hand, strategies such as combination therapy of anti-PD-1 immunotherapy and radiotherapy in this case that make patients with limited treatment options benefit from immunotherapy might be applicated at present.

The possible mechanisms of the synergistic anti-tumor effect of combination therapy have been investigated by many researchers so far. We summarized them as follows, including tumor burden reduction, immunity activation, and tumor microenvironment modification. First, radiotherapy could reduce the tumor burden and create a background of minimum tumor burden for immunotherapy. Second, radiotherapy can fully trigger the recognition of tumor cells by antigen-presenting cells. Irradiation can directly destroy the DNA, allowing more neoantigens released by tumor cells to trigger immune responses (14). Some innate immune pathways can be activated during radiotherapy to regulate the anti-tumor immune responses. Irradiation-induced cGAS-STING pathways can lead to the recruitment of dendritic cells and trigger the type I IFN signaling, thus regulating the adaptive immune response and reinforcing the cytotoxic T cells (15). Third, radiotherapy can modify the tumor microenvironment, potentially affecting the immune compositions, and priming the adaptive immunity. Localized irradiation can induce chemokines involved in the recruitment of effector T cells, converting the tumors into tissues susceptible to immune attack (16). In our case, the primary tumor had significantly high infiltration of M2 macrophages, which contributed to the immunosuppressive tumor microenvironment. Klug et al. have recently shown that low doses of radiotherapy can reprogram tumor-associated macrophages to a M1 phenotype, which conversely enhanced the efficacy of adaptive immunity (17). Probably, the macrophages of the primary and metastatic tumors in this patient had experienced such a conversation from M2 to M1 under irradiation, initiating a significant change in the tumor immune microenvironment, which deserves further studies and clinical trials on the dynamic evolution of ICC under combined therapy.

Currently, chemotherapy such as gemcitabine plus cisplatin is considered as the only first-line treatment for metastatic ICC. However, the chemotherapy regimen results in a severe (grade 3 or 4) toxic effect rate of about 70% (1). Due to the toxicities of traditional chemotherapeutic drugs, good performance status is often required for chemotherapy. But in fact a large number of advanced-stage patients have bad performance status, so that they are intolerable to chemotherapy. On the contrary, immunotherapy combined with radiotherapy has relatively slighter short-term side effects and may be more suitable for these patients. Clinical trials that investigated the possibility of anti-PD-1 immunotherapy combined with radiotherapy as first-line treatment in ICC patients could be conducted.

During the treatment, new lung lesions occurred while the other lesions demonstrated controlled, which indicated different treatment responses across organs, namely a mixed response. This atypical response pattern has been noticed in previous studies (18, 19). According to the conventional radiological response criteria, the Response Evaluation Criteria in Solid Tumors (RECIST) version 1.1, this would be evaluated as PD. However, patients with the response pattern were found to have non-inferior OS compared with those who had controlled diseases, which means the RECIST underestimates the clinical benefit of immune checkpoint blockade. Thus, several novel response evaluation criteria have been proposed recently, including the iRECIST, the immune-related response criteria (irRC), and the immune-modified RECIST (imRECIST). According to these criteria, the patient in this case should not be characterized as PD in the situation, and the combination therapy could be continued, which was proven to be a sensible choice afterwards.

## Conclusions

In conclusion, we analyzed the most valuable biomarkers for immunotherapy response and demonstrated their complexity and inconsistency in an ICC patient who had limited treatment options. The current dilemma made us adopt the combination therapy of anti-PD-1 immunotherapy and radiotherapy as his first-line treatment, which led to a complete response and prolonged survival time. This suggests their synergic anti-tumor effect and the bright prospect of combination therapy. Further efforts are required to investigate the combination therapy in ICC patients.

## Data Availability Statement

All datasets generated for this study are included in the article/supplementary material.

## Ethics Statement

Ethical review and approval was not required for the study on human participants in accordance with the local legislation and institutional requirements. The patients/participants provided their written informed consent to participate in this study. Written informed consent was obtained from the individual(s) for the publication of any potentially identifiable images or data included in this article.

## Author Contributions

Z-LL, XL, and HP drafted the manuscript and performed data analysis. Z-WP, DT, and SP were involved in manuscript editing. J-TL, YB, and MK treated the patient and designed the study. All authors read and approved the final manuscript.

## Conflict of Interest

The authors declare that the research was conducted in the absence of any commercial or financial relationships that could be construed as a potential conflict of interest.
